# Exploring patients’ perceptions and experiences of treatments for the prevention of variceal bleeding: a qualitative study

**DOI:** 10.1136/bmjgast-2021-000684

**Published:** 2021-08-16

**Authors:** Chris Poyner, Dhiraj Tripathi, Jonathan Mathers

**Affiliations:** 1 Institute of Applied Health Research, University of Birmingham, Birmingham, Birmingham, UK; 2 University of Birmingham, Birmingham, UK; 3 Liver Unit, Queen Elizabeth Hospital, Birmingham, Birmingham, UK

**Keywords:** liver cirrhosis, portal hypertension, endoscopic procedures, oesophageal varices

## Abstract

**Background:**

The most common fatal complication of liver cirrhosis is haemorrhaging caused by variceal rupture. The prevention of the first variceal bleed is, therefore, an important clinical goal. Little is known about patients’ experience of treatments geared towards this, or of their perceptions of treatments prior to being exposed to them.

**Aims:**

To explore the factors impacting patient preference for, and actual experience of carvedilol and variceal band ligation.

**Methods:**

Semistructured interviews were conducted with 30 patients from across the UK at baseline, prior to random allocation to either carvedilol or variceal band ligation. Twenty patients were interviewed a second time at 6-month follow-up. Five patients who declined the trial were also interviewed. Data were analysed using thematic analysis.

**Results:**

There was no clear preference for either treatment pathway at baseline. Key factors reported by patients to influence their treatment preference included: negative experiences with key treatment processes; how long-term or short-term treatment was perceived to be; treatment misconceptions; concerns around polypharmacy and worries around treatment adherence. Patient treatment experience was influenced by their perceptions of treatment effectiveness; clinical surveillance; clinician interaction and communication, or lack thereof. Carvedilol-specific experience was also influenced by the manifestation of side effects and patient dosage routine. Variceal band ligation-specific experience was positively influenced by the use of sedation, and negatively influenced by the procedure recovery period.

**Conclusions:**

These data do not support a view that the patient experience of beta-blockade for prevention of variceal bleeds is likely to be superior to variceal band ligation.

**Summary box**

**What is already known about this subject?**

- The relative clinical effectiveness of competing treatments (carvedilol vs variceal band ligation), for the primary prevention of variceal bleeds is contested.
- There is limited quantitative evidence from the USA suggesting that both treatment-naïve patients and clinicians are more likely to state a preference for variceal band ligation as first-line treatment.
- To date, there have not been any qualitative research studies that have examined cirrhotic patients’ views and experiences of treatments for the prevention of variceal bleeding.

**What are the new findings?**

- The preferences of treatment-naïve patients are varied.
- Patient perceptions of variceal band ligation were positively influenced by their experience of this treatment pathway.
- The patient experience of beta-blockade for prevention of variceal bleeds is not likely to be superior to variceal band ligation.

**How might it impact on clinical practice in the foreseeable future?**

- Ensuring patients are fully informed of the nature of treatments may improve their experience of treatment processes and also improve adherence.
- Our findings may help to inform clinical decision making when considering treatment options for patients with medium to large varices.

## Introduction

Estimates suggest that globally liver cirrhosis is responsible for 1.16 million deaths annually.^1^ In the UK, cases have risen from 6999 in 2012 to 7630 in 2016, an increase of 8.3%.^2^ Liver cirrhosis is the fifth largest cause of death^3^ and the third largest cause of premature death in the UK.^4^ The lived experience of liver cirrhosis, often understood using qualitative research methods, is poorly evidenced, with patient perceptions of treatments representing a key gap.^5^ Much of the qualitative research evidence that does exist relates to patients eligible for liver transplantation, for example, examining patients’ experiences pretransplantation^6 7^ and post-transplantation.^8–10^ These patients are not necessarily representative of the wider liver cirrhosis patient population, with most ineligible for a transplant.^11^

A major common complication of liver cirrhosis is oesophageal varices, due to portal hypertension. At diagnosis approximately 30% of patients have oesophageal varices, rising to 90% after 10 years.^12 13^ At least 3000 patients are admitted to hospital in England per year with variceal bleeding, with inpatient mortality of 15% and 1-year mortality of up to 40%.^14^ The prevention of the first variceal bleed is therefore an important clinical goal.

Treatments aiming to prevent variceal bleeds include non-selective beta blockers, such as carvedilol, and/or variceal band ligation (VBL).^15 16^ Currently, there is inconsistency within best practice guidance in the UK, with the British Society of Gastroenterology recommending a non-selective beta-blocker as first-line therapy,^15^ and the National Institute for Health and Care Excellence (NICE) recommending VBL.^13^ Although at present the NICE guideline is under review, the relative clinical effectiveness of each treatment remains contested.

There is limited quantitative evidence from the USA suggesting that both treatment-naïve patients and clinicians are more likely to state a preference for VBL as first-line treatment, despite the predominance of beta-blockade in practice.^17^ To date, however, there have not been any qualitative research studies that have examined cirrhotic patients’ views and experiences of treatments in depth. Information relating to patients’ treatment preferences and the acceptability of treatment options provides a crucial adjunct to evidence of the clinical efficacy of treatment options in clinical decision making. The carvedilol versus variceal band ligation in primary prevention of variceal bleeding in liver cirrhosis study (CALIBRE) is a randomised controlled trial (RCT) investigating the effectiveness of carvedilol versus VBL for the prevention of variceal bleeds.^14^ This paper presents findings from the qualitative research component of the CALIBRE study which aimed first to explore patients’ preferences for treatment, and second to describe their actual experience of VBL and carvedilol.^14^

## Materials and methods

### Study design

This qualitative research study was conducted during an internal pilot trial of the CALIBRE study, a multicentre RCT comparing carvedilol and VBL for the primary prevention of variceal bleeds from medium and large varices.

To meet the inclusion criteria for CALIBRE patients need to have been assessed by a consultant gastroenterologist as having medium (grade II) or large (grade III) varices as defined in the British Society of Gastroenterology guidelines^15^ that have never bled.

### Sampling and recruitment

For the qualitative research study, we sampled patients randomised to carvedilol and VBL, as well as those who were eligible but declined to participate in CALIBRE. Potential participants were identified by research teams recruiting to CALIBRE at hospital sites in England, Scotland and Wales and provided with written information about the qualitative study. Patients expressing an interest in participation were consented to provide contact details to the qualitative research team at the University of Birmingham, UK. A qualitative researcher (CP) then contacted patients to answer any questions and to arrange a convenient date and time for interview. Audiorecorded consent was taken prior to interview.

### Data collection

Semistructured one-to-one interviews provide rich data relating to individuals’ perspectives and lived experience of disease and treatments. Patients randomised to carvedilol and VBL were invited to interview at two time points; within 1 month of randomisation and after 6 months of trial participation. At index interview ten of the 15 VBL patients had experienced their first procedure. All 15 carvedilol patients had commenced treatment. Baseline interviews with patients were conducted between February and September 2019. Follow-up interviews were conducted between August and March 2020. Patients declining participation in CALIBRE took part in a one-off interview. The majority of interviews were conducted over the phone (n=52), with a small number (n=3) conducted face to face in a private room at the University of Birmingham. Prior to data collection, it was estimated that interviews with approximately 15 patients in each trial group would allow saturation of core analytical themes.

Interview schedules guided interview conduct and were refined iteratively following initial interviews (see online supplemental file A). Baseline interviews with trial participants focused primarily on the acceptability of CALIBRE, exploring patients’ perspectives on the recruitment process and their treatment preferences prior to randomisation. To contextualise these perspectives participants were also asked to briefly discuss their prior experience of liver cirrhosis and its impact on daily life. Follow-up interviews focused on patients’ experience of treatment with carvedilol or VBL. Topics for discussion included the occurrence of side effects and their impact on health and quality of life, the experience of undergoing treatment (eg, medication routines, the VBL procedure and recovery) and how patients felt about the diagnosis of varices and the risk of bleed following treatment. Prior to the follow-up interview, the interviewer (CP) reread the transcript of the initial interview, and noted additional areas for follow-up with the interviewee based on prior discussion. Interviews with patients declining the trial focused on their reasons for this decision, including associated treatment preferences.

10.1136/bmjgast-2021-000684.supp1 Supplementary data

Interviews were audiorecorded and transcribed clean verbatim by a professional transcription company. Data were analysed with reference to interview transcripts, recordings and notes taken during and after interview. Data collection and analysis proceeded iteratively. At the point at which initial sample estimates for baseline interviews were attained, it was judged that data saturation of core analytic content had been achieved and recruitment to the qualitative study was ceased.^18^

At follow-up, it was judged that saturation pertaining to patients’ experience of treatment for varices had also been attained.

### Data analysis

An inductive thematic analysis informed by framework was undertaken.^19^ Each transcript was read multiple times to aid familiarisation, and each recording was listened back to check the accuracy of the transcript. A sample of transcripts were independently coded by CP and JM, then discussed. This process informed further iterative coding. Codes were grouped into categories and all data were indexed. Relationships between categories were examined in order to interpret overarching themes. Preliminary descriptive analytical accounts were shared between CP and JM to aid this process. Emerging interpretations were discussed among the broader research team. Associative analysis, comparing the accounts of patients allocated to VBL with those allocated to carvedilol, was undertaken. Data were managed via Nvivo qualitative data analysis software (V.12).

## Results

Thirty trial participants were interviewed at baseline (table 1). Of these, 20 were available for interview at follow-up. Of the 30, 15 were allocated to carvedilol (10 followed up) and 15 to VBL (10 followed up); 17 were male and the median age was 60 (range 27–80). Five patients who declined consent to CALIBRE were also interviewed.

**Table 1:** Sample characteristics of interviewees who were CALIBRE trial participants

Treatment	Carvedilol	15 (50%)
	VBL	15 (50%)
Presence of hepatic decompensation	Yes	4 (13.33%)
	No	26 (86.66%)
Alcohol-related liver disease	Yes	10 (33.33%)
	No	20 (66.66%)
Grade of varices	Grade 2	26 (13.33%)
	Grade 3	4 (86.66%)
Age at randomisation	18–50 years	7 (23.33%)
	51–70 years	19 (63.33%)
	>70 years	4 (13.33%)
	Median=60	
Gender	Male	17 (56.66%)
	Female	13 (43.33%)
Country	England	23 (76.66%)
	Scotland	6 (20.00%)
	Wales	1 (03.33%)

VBL, variceal band ligation.

### Themes

Findings are presented in three sections. First, data related to interviewees’ treatment preferences at baseline, prior to randomisation, are reported. Next, patients’ actual experience of treatment at follow-up are described. Finally, the ‘peace of mind’ afforded to patients by treatment is considered. Within each section, findings related to carvedilol and VBL are presented separately. Themes, subthemes and findings are summarised in table 2.

**Table 2:** Summary table of themes, subthemes and findings

Theme	Treatment	Subtheme	Summary findings
Treatment preferences at baseline interview	Carvedilol	Aversion to gastroscopic procedures	All patients had experienced at least one gastroscopy previously and preference for carvedilol was often influenced by this experience.
	Carvedilol	Misconceptions around the nature of the VBL procedure	Some patients described VBL inaccurately, that is, as surgery, or not requiring sedation to justify their preference for carvedilol.
	Carvedilol	The causal mechanism of carvedilol	A small number of patients mentioned the causal mechanism of carvedilol as the key reason for their stated preference.
	VBL	Concern about polypharmacy	Some patients described already taking several medications as a concern, or a more general dislike of taking tablets
	VBL	Carvedilol adherence	Some participants went on to report a concern about their capacity to remember to take carvedilol on a daily basis
	VBL	History of mental health challenges	In a small number of cases, patients with a history of mental health issues were concerned that taking medication could cause such issues to resurface
	VBL	VBL as the quicker solution	Some participants perceived VBL as a quicker solution due to the long-term nature of carvedilol prescription, and a perception that the issue of variceal bleeding is dealt with once varices have been banded
	VBL	Lower blood pressure as problematic	A small number of patients felt that having their blood pressure lowered would negatively impact their health or quality of life due to a fear of blacking out or fainting.
Reasons given for a stated lack of treatment preference	Neither	Lack of knowledge on which to base a preference	Slightly fewer than half of the trial participants interviewed reported no clear treatment preference at the baseline interview. Most commonly, patients who did not have a clear preference were most concerned by overall health outcomes.
Patients’ actual experience of treatment	Carvedilol	Side effects	A lack of side effects contributed to satisfaction with treatment as did a perception of improved health outcomes. A slight majority of participants did report at least one negative side effect. Reported side effects included: dizziness, fainting, nausea, diarrhoea, palpitations, fatigue and low mood. The most commonly discussed side effect was fatigue.
	Carvedilol	Importance of dosage routine to positive experience of carvedilol	For some patients, side effects lessened or were alleviated after the first month or so of taking the medication. Dosage routine alterations were often key to this.
	VBL	Sedation as key to a better patient experience than anticipated	Patients who described anxiety around VBL due to previous negative experiences of endoscopy, reported a better than anticipated experience due to the positive impact of sedation.
	VBL	Trust in clinician conducting procedure	Treatment acceptability was reported as low by patients where they were not confident in the clinician performing the procedure. In many cases patients suggested that trusting relationships with their consultants were key to a positive VBL procedure experience
	VBL	Pain during VBL recovery period	The recovery from VBL was reported as the most challenging aspect. Pain and nausea were reported. Patients described the need to eat, soft and cold foods. Some felt underprepared for their recovery.
	Both	Peace of mind related to perceptions of treatment effectiveness	In most cases, carvedilol patients expressed concern regarding a lack of continued monitoring and desired reassurance that the treatment was working. For VBL patients, a key positive of the experience was what was perceived as knowledge that their varices had been, or were in the process of being eradicated.

VBL, variceal band ligation.

Data from interviews with patients declining the trial are included in the first section as they further illustrate treatment preferences for patients who are treatment-naïve.

### Treatment preferences at baseline interview

Among the patients participating in the pilot trial there was no clear preference for either treatment. Broadly these interviewees were evenly split between those with a strong preference for carvedilol or VBL, and those without a strong preference for either treatment. Patients declining the trial all expressed an aversion to gastroscopy and hence VBL. Patients’ reasoning behind stated preferences are detailed below.

### Reasons given for a stated preference to be allocated to carvedilol

#### An aversion to gastroscopic procedure

In many cases, for those with a preference for carvedilol, an aversion to gastroscopy was key, rather than any perceived benefits associated with carvedilol. All patients had experienced at least one gastroscopy previously and preference for carvedilol was often influenced by this experience. Distressing previous gastroscopies were commonly reported:

It was pretty horrendous and I was aware I was choking all the way through, it was horrible. So I thought please let it be the other one… (Interviewee 3)

Patients declining to participate in CALIBRE all described this as a key factor in the decision to do so, not wanting to be allocated to VBL:

I went I am not doing it. I am not going to have an endoscopy every month. (Decliner Interviewee D3)

#### Misconceptions around the nature of the VBL procedure

Some apparent misconceptions about the nature and detail of VBL seem to have impacted on preferences and acceptability to some patients. One case was clearly demonstrative of this:

As I say my GP… gave me a printout… obviously when you have the banding you have to be put out… I might be strong enough to be put out. So I don’t know whether it’s going to go ahead. (Interviewee 27)

Here, the patient with a planned VBL trial procedure discussed a consultation with their general practitioner. This patient had had a preoperative assessment for a surgical procedure that had raised concerns about their ability to tolerate a general anaesthetic. Their anxiety related to VBL appeared largely to be based on an incorrect interpretation of ‘VBL as surgery’, thereby requiring a general anaesthetic. Indeed, another patient mentioned how the explanation of VBL as *‘*not cutting me up*’* was key in informing their consent:

… if they had said there’s a bit of extra cutting me or procedures like that… I would have probably said no… because everything was explained to me, that was fine. (Interviewee 15)

Other patients reported being unaware VBL was performed under sedation. Indeed, one patient reported not knowing about the sedation until being told by a clinician just prior to their VBL procedure:

I always just have the throat spray for endoscope, but when I got into the room they said oh no for the banding you have to be sedated. (Interviewee 23)

#### The causal mechanism of carvedilol

As mentioned, an aversion to elements of VBL was key, rather than any perceived benefit of taking carvedilol itself. Despite this, a small number of patients mentioned the causal mechanism of carvedilol as the key reason for their stated preference:

I reasoned… the beta blockers were doing something to all of the veins, and it seemed to me more logic, but then that’s an engineer talking. (Interviewee 26)

### Reasons given for a stated preference to be allocated to VBL

#### Concern about polypharmacy

Similar to carvedilol, preferences for VBL were often associated with concerns regarding the alternate treatment rather than a positive view of VBL. Some patients described already taking several medications as a concern, or a more general dislike of taking tablets:

I am taking enough tablets and everything, so… I would rather have the banding, it’s dead simple, get it over and done with… (Interviewee 16)

#### Carvedilol adherence

Some participants went on to report a concern about their capacity to remember to take carvedilol on a daily basis:

I struggle to keep up to date with them, because it’s not as if I can take them all at the one, there’s a couple I take at lunchtime and the other ones to be taken at night, so I do get confused some days… (Interviewee 12)

Here, the focus was adherence in the context of polypharmacy. The number of tablets was not the only issue. The daily timing of medications was also highlighted. There were points during other interviews where respondents mentioned a failure to adhere strictly to taking the required carvedilol dose due to human error.

#### History of mental health challenges

In a small number of cases, patients with a history of mental health issues were concerned that taking medication could cause such issues to resurface:

… because of my history… [of mental health issues] I would have preferred the banding. (Interviewee 8)

#### VBL as the quicker solution

Some participants perceived VBL as a quicker solution to varices than carvedilol, due to the long-term nature of carvedilol prescription, and a perception that the issue of variceal bleeding is dealt with once varices have been banded. This was often reinforced by an aversion to taking tablets:

I am not really the keenest person for taking tablets and things like that so being sedated for 10/15 minutes and the job is done sort of thing, which it was basically. (Interviewee 1)

#### Lower blood pressure as problematic

A small number of patients felt that having their blood pressure lowered would negatively impact their health or quality of life due to a fear of blacking out or fainting. Lowered blood pressure was therefore perceived by some as a negative result of taking carvedilol despite the positive impact this may have on the likelihood of a variceal bleed:

…lowering your blood pressure and all that, and I am thinking that could just be causing more problems. (Interviewee 4)

### Reasons given for a stated lack of treatment preference

#### Lack of knowledge on which to base a preference

Slightly fewer than half of the trial participants interviewed reported no clear treatment preference at the baseline interview. Most commonly, patients who did not have a clear preference were most concerned by overall health outcomes, and as Interviewee 29 described, they had no basis to think that one treatment was superior to the other:

No, not really, no. I’ve got no basis… so just on the back of the clinicians. (Interviewee 29)

### Patients’ actual experience of treatment—follow up interviews

#### Carvedilol

##### Side effects

Some patients did not report any side effects:

…there’s been no side effects from taking them anyhow, so it’s like I am quite happy at the moment with that. (Interviewee 14)

A lack of side effects contributed to satisfaction with treatment as did a perception of improved health outcomes, such as lowered blood pressure:

So I am happier… it’s brought my blood pressure down. (Interviewee 5)

However, a slight majority of participants did report at least one negative side effect, at least when initially taking the full recommended dose of carvedilol. Reported side effects included: dizziness, fainting, nausea, diarrhoea, palpitations, fatigue and low mood. Weight gain, chest pain and breathlessness were also reported.

The most commonly discussed side effect was fatigue. Interviewees often identified fatigue as a pre-existent issue, but felt that their carvedilol dose was exacerbating it:

No, I read the things, side effects I probably am a little more tired than I used to be, mind you part of that is age. (Interviewee 21)

Dizziness was also discussed by multiple participants and was not perceived to be a pre-existing issue:

But probably the first thing I got a bit dizzy. (Interviewee 15)

Despite patients reporting several different side effects, some pointed out how it was challenging to pinpoint carvedilol as the cause in the context of polypharmacy or multiple comorbidities:

Relatively recent [start of muscle fatigue]… the weakness does seem to have increased. Again whether that’s got anything to do with the trial or the drug I don’t know… (Interviewee 26)

A small number of interviewees reported concerns about side effects to the point of having thought about crossover or withdrawal:

At first I did, yeah, I am not going to lie to you, when I was getting a bit dizzy and what have you, I thought maybe it’s not for me this… (Interviewee 28)

In the case of interviewee 28, a dosage reduction from the full 12.5 mg dose to 6.25 mg daily, was key in facilitating their continuation. However, despite having their dosage reduced, interviewee 24 felt they would be unable to tolerate carvedilol beyond the trial:

If this is it and they feel this [dizziness and headaches] is definitely a side effect of the carvedilol then I would say well I need to try something else. (Interviewee 24)

#### Importance of dosage routine to positive experience of carvedilol

For some patients, side effects lessened or were alleviated after the first month or so of taking the medication. The following interviewee described altering their dosage routine after their 4-week trial follow-up:

When I was first taking it I was taking it in the morning and you could guarantee about two or three hours after I had taken it I came out in a hot sweat, felt faint, felt dizzy. I now take it at night and I’ve overcome that. (Interviewee 5)

Routine was also described as key to adherence. In most cases patients described having little to no issue with remembering to take their carvedilol on a daily basis as a result of strictly keeping to their medication regimen:

Fine, I’ve now got my routine, I have my breakfast and then take the pill. (Interviewee 8)

#### Variceal band ligation

##### Sedation as key to a better patient experience than anticipated

While some interviewees talked about being nervous in anticipation of VBL, due to poor experiences of previous endoscopies, most described how their actual experience challenged this preconception. For example, Interviewee 9 described the whole experience as ‘smooth’:

[VBL was] Smooth as in I really don’t really remember much about it at all, sedation as I said it was excellent. (Interviewee 9)

The pain and discomfort patients described during investigative endoscopies, when without sedation, was not an issue in the majority of cases. Interviewee 23 made the comparison between the sensation of gastroscopy without sedation, and the VBL with sedation, suggesting they felt less during their VBL procedure:

… because they do sedate you, you don’t feel quite as much, because before my endoscopies I’ve never been sedated, I’ve always had them without sedation. (Interviewee 23)

Another interviewee mentioned how they had anticipated feeling the VBL procedure being performed, but were surprised when this was not the case:

Even when they did the banding I never felt a thing, because I thought I would feel it but no never felt a thing. (Interviewee 16)

#### Trust in clinician conducting procedure

Treatment acceptability was reported as low by patients where they were not confident in the clinician performing the procedure, for example, if a registrar performed a VBL procedure instead of the patient’s consultant. A small number of patients experienced this, with one stating that they would consider withdrawal if their consultant did not agree to conduct future procedures:

I stated that if I didn’t get the same person doing the procedure I wasn’t carrying on with it, because I really had real confidence in him. (Interviewee 9)

In many cases patients suggested that trusting relationships with their consultants were key to the VBL procedure experience:

I think it’s been the relationship I’ve got with the consultant obviously… I think the main thing has been just feeling comfortable doing it [VBL procedure]. (Interviewee 16)

#### Pain during VBL recovery period

The recovery from VBL was consistently reported as the most challenging aspect. Postprocedure, interviewees described issues with pain, nausea, discomfort and swallowing:

Sometimes I think it’s how much it’s banded, but sometimes can be fine, other times can take ten days before I can eat properly, and very painful, I didn’t really expect that. (Interviewee 6)

Interviewee 6 reported a variable length of time to fully recover and begin eating normally again. They hypothesised that this was associated with the number of bands being attached during a session. Intense pain was also reported by some patients:

Sometimes at night I would wake up and it feels like someone has kicked me in the gut so to speak, and it feels very bruised and you feel like your body is full of acid, and you can feel quite sick. (Interviewee 19)

All patients who experienced pain during recovery described the need to carefully select specific types of food to eat, such as soft and cold foods including yoghurt, ice cream and some cereals:

… since the procedure was done just had a soft cold diet… I literally could only tolerate products, Weetabix, that sort of food. (Interviewee 17)

Although patients reported knowing that they would need to recover from the VBL procedure, which may involve some pain and short term dietary alterations, the period of recovery was underestimated by some. This caused some issues for patients in terms of their diet, and/or needing time off work:

I ended up being off for a whole week because for me it was quite painful once it was done. (Interviewee 1)

Interviewees seemed unaware of this prior to the procedure suggesting a lack of discussion with clinicians concerning the duration of the post VBL recovery period.

### Peace of mind related to perceptions of treatment effectiveness

#### Carvedilol

One aspect that seemed to influence the experience of treatments was how confident patients’ felt about the effectiveness of their treatment. In most cases, patients allocated to carvedilol expressed some concern regarding a lack of continued monitoring and desired reassurance that the treatment was working for them via endoscopic surveillance, despite some interviewees having stated a preference for carvedilol to avoid additional endoscopic procedures:

This is a question I asked, that how do I know if this is working… I was surprised that there’s no [gastroscopy] not that I relished them because they’re a horrible procedure… (Interviewee 24)

#### Variceal band ligation

For patients allocated to the VBL pathway, a key positive of the experience was what was perceived as knowledge that their varices had been, or were in the process of being eradicated, meaning they were no longer a concern:

… I actually received a letter from the clinic saying they have been obliterated so that’s good from that point of view. (Interviewee 17)

Here, VBL was perceived as highly effective as varices are no longer ‘there’ or have been ‘obliterated’. Patients seemed to have gained peace of mind and reassurance as a result, something that patients allocated to carvedilol did not achieve. Peace of mind was also associated with consultant feedback received by patients allocated to VBL. Final endoscopic confirmation at the end of the banding process appeared to help confirm the effectiveness of the procedure in the patient’s mind, securing a sense of confidence in the treatment pathway:

I went back for my last [VBL session] … the doctor said, ‘there’s nothing else to band.’… well that was great for me. (Interviewee 6)

## Discussion

While overall there was no clear preference for VBL or carvedilol among patients consenting to the CALIBRE pilot trial, some hoped to be allocated to carvedilol in order to avoid VBL and vice versa. Previous research has suggested a preference for VBL in treatment-naïve patients who receive detailed information about procedures and potential side effects, due to concerns around hypotension and shortness of breath.^17^ Our findings support the premise that patient preferences for treatment of varices are often underpinned by perceptions of potential negative outcomes associated with their least preferred option. Patients who expressed a treatment preference tended to rationalise this by highlighting fears held about taking carvedilol (due to low blood pressure, concerns around polypharmacy and issues with adherence) or undergoing a VBL procedure. All of the interviewees who declined the trial also expressed an aversion to VBL. Some of these views were influenced by clear misinterpretations of the procedure, such as perceiving VBL as surgery. However, most often patients were concerned VBL would mirror previous distressing experiences of gastroscopy.

Memories of previous endoscopies have been found to shape perceptions of endoscopies going forward^20 21^ and induce feelings of fear or nervousness in patients faced with the prospect of further endoscopies. Previous qualitative research also suggests that experience of gastroscopy influences future patient adherence.^22^ This highlights the importance of clear communication with patients that sets out the experiential differences between VBL and non-sedated gastroscopy, described by the patients in this study. Ensuring patients’ experiences of gastroscopies are as comfortable as possible may also contribute to alleviating VBL procedure concerns for some patients. As evidenced by follow-up interviews with patients allocated to VBL, the use of sedation clearly delineates the VBL procedure experientially from routine diagnostic gastroscopy. Many expressed surprise at the relative comfort of the banding procedure. Post hoc the recovery period and accurate information concerning that were far more significant for patients than the experience of the procedure itself. In some cases, patients felt underprepared for the period of recovery and underestimated the duration that issues, such as pain, would last. A small number of patients related the trust they had in the clinician performing the procedure, to the quality of the procedure experience and the nature and duration of the recovery period. It is hoped our findings hold implications for, and will be of value to, those recruiting patients into the CALIBRE trial. These findings have been communicated through internal reports and guidance materials.

While acknowledging that this research cannot provide binary views of which treatment is better experientially, these qualitative data suggest that the actual (in practice) experience for patients allocated to VBL is relatively favourable. Patients allocated to both groups on the whole expressed satisfaction with trial treatments, but the side effects of carvedilol were a significant issue for some patients, as was adherence. Side effects reportedly decreased in severity or ceased due to patient adaptation after continued exposure and/or alterations to the dosage routine. Most interviewees appeared to be adhering to their prescribed carvedilol regime, although a small number stated that they were not taking carvedilol or were taking it intermittently without reporting this to clinicians. Moreover, some patients described being doubtful of their will to continue their carvedilol prescription beyond the 12-month trial follow-up. Although this is the first qualitative study to explore the experience of patients with cirrhosis prescribed beta blockers, challenges with medication regimes have been shown to reduce the likelihood of adherence in patients with heart disease.^23 24^ Side effects were the most important barrier to adherence, with other contributory factors including polypharmacy, healthcare system barriers and comorbidities.^23 24^

An important component of perceived treatment effectiveness that patient interviewees spoke about, was the peace of mind treatments for varices could afford them, especially considering the potentially grave outcomes of a variceal bleed. ‘End-of-treatment’ reassurance of the eradication of varices contributed to greater reassurance on the part of patients allocated to VBL. Our data also suggest the therapeutic relationships VBL patients have with their consultant, due to repeat VBL sessions, may also positively influence perceptions of treatment effectiveness. This was a key reason for patient satisfaction expressed with the VBL treatment pathway, while the lack of clinical engagement was of concern to many carvedilol patients. Other studies have shown that clinicians are crucial in fostering patients’ engagement along all the phases of treatment processes, including patient adherence to medication regimes.^25^ Research in cirrhosis suggest that communication with health professionals can influence patients’ mental and physical well-being, that patients can be poorly informed about the severity of their condition and their future health needs,^6 26 27^ resulting in unmet emotional needs among this patient group.^28^

The in-depth qualitative approach, involving interviews at two time points, enabled the collection of rich accounts of patient treatment preferences and their post hoc reported experience of those treatments. Thus, we have been able to observe how patients’ perceptions of the treatments were influenced over time by their experience (both positively and negatively) and understand how this experience could potentially be improved for patients with medium to large varices. The depth and quality of the data collected was enhanced due to the interviewer being a qualitative researcher, independent of the patients’ medical care. Patients were able to discuss their experiences openly, safe in the knowledge that discussions would not impact on their care. The interview dataset comprises 55 in-depth interviews and data saturation regarding views and experiences of VBL and carvedilol was judged to be attained within this sample, with a balance between patients allocated to carvedilol and VBL. Patients were recruited from sites across the UK.

However, it should be noted that the study made use of a sample derived from a clinical trial. Some patients with strong feelings towards either treatment, may have refused participation outright, to choose their preferred treatment outside of the trial context. Indeed, the small number of those we interviewed who declined the trial, cited an aversion to the VBL pathway as a key factor in their decision. Thus, we cannot make claims regarding the prevalence of pre-treatment views of VBL relative to carvedilol in the general population of patients with cirrhosis and indeed that is not the aim of qualitative studies such as this. Five of the VBL patients interviewed had no treatment exposure prior to index interview, whereas all carvedilol patients had begun treatment at this point. It is possible that initial experiences of treatment may have influenced how initial preferences were reported. Of the 30 patients interviewed at baseline, 20 completed a follow-up interview. In the majority of cases (n=7) drop-out was due to participants being uncontactable at follow-up. However, one participant refused a follow-up interview, and two others had withdrawn from the pilot trial. Drop-out was evenly distributed across both treatment groups, suggesting this was not associated with treatment. Key themes related to the experience of both treatments were consistently recounted by participants in follow-up interviews.

## Conclusion

In this study, we have described the views of patients with medium to large varices on VBL or carvedilol, for primary prevention of variceal bleeding, both before and after treatment exposure. Our findings demonstrate that the preferences of treatment-naïve patients are varied, with some stating a preference for either carvedilol or VBL, and some having no firm preference. On the whole, patient perceptions of VBL were positively influenced by their experience of this treatment pathway. Despite this, many patients in the VBL arm described longer than expected recovery periods during interview and it would appear that some patients in the carvedilol arm may have benefitted from guidance regarding side effect management (eg, timing of dose). Our findings hold important implications for clinical practice, including the discussions which clinicians hold with their patients about treatment options. Further research examining the effective communication of patients’ experiences of these treatments is warranted. Ensuring patients are fully informed of the nature of treatments may improve their experience of treatment processes and also improve adherence. Further evidence related to the effectiveness of VBL versus carvedilol in the primary prevention of variceal bleeds is required. This is of particular importance in a post-COVID-19 context, whereby the use of carvedilol may become more prevalent, for example, if emergency guidance for carvedilol use (such as that issued by the British Society of Gastroenterology)^29^ influences postpandemic use. These data do not support a view that the patient experience of beta-blockade for prevention of variceal bleeds is likely to be superior to VBL.

## Data Availability

Data are available on reasonable request. The data are not in a repository at present. The data are securely held in an encrypted University of Birmingham server, accessible by Chris Poyner Orchid ID: 0000-0001-6271-2626.
