# Supplementary material for: Exploring patients’ perceptions and experiences of treatments for the prevention of variceal bleeding: a qualitative study
Source: BMJ Open Gastroenterol. 2021 Aug 16;8(1):e000684. doi: 10.1136/bmjgast-2021-000684 (PMC8370546; doi:10.1136/bmjgast-2021-000684)
Supplement: Supplementary data [file bmjgast-2021-000684supp001.pdf]

Supplemental A

Interview Schedule- CALIBRE Phase 1. Patients who have consented to trial, shortly after randomisation

Introduction and opening remarks

1.) Introduce self and tell participant you will begin recording
2.) Achieve informed consent verbally, take participant through information sheet and consent form, ensuring participant is aware of the broad aims of the study, and their rights as research participants. Fill in consent form on their behalf (if telephone interview). Keep a copy on file and post a copy to participant.
3.) Explain the value of the voice and experience of the participant to the aims of the study.
4.) Ensure the participant is comfortable to begin the interview and give them the opportunity to ask any questions before beginning.

Background of participant, context

1.) May I first ask how old you are?
2.) Can you tell me a bit about your personal background? - Probe around where from originally and react to what information participant divulges.
3.) Are you currently working?
4.) Who are the important people in your life? - Probe around family, friends if required.
5.) When did you first start to notice there may be issues with your health?
6.) What were your initial symptoms? How did they develop?
7.) Did these symptoms impact upon your daily life? If so at what point and how? – Probe around social life, relationships, working life, activities of daily living
8.) At what point did you decide to seek medical help? – Probe around time period since initial symptoms, time relevant to present day and possible catalyst/motivation for seeking help.
9.) What helped you make the decision to seek medical help?
10.) At what point were you formally diagnosed with LC?

Recruitment, participation and trial process

1.) How did you become interested in taking part in this study? – Probe around how they found out about the study and what motivated them to take part (Family advice, Clinician advice and/or Personal beliefs)
2.) Who introduced you to the study?
3.) What were you told about the study at the point of introduction?
4.) Was this information useful?
5.) Were you supplied with 2 information sheets for the study?
6.) At what point did you read the PIS(s)?
7.) What did you think about the PIS(s) – Probe around understanding, accessibility, size and language used
8.) Are there any ways you feel we could improve the PIS(s)?
9.) Do you feel you were presented with enough information, to understand the study and what it, involves prior to agreeing to participate? Was there anything you were unsure about at this point?
10.) Do you feel you had enough time to fully understand what the study involves before agreeing to participate?
11.) Was it a difficult decision to agree to take part in the study? – Probe around length of time taken to agree to participate and influence of close friends/family in decision making process

Supplemental A

12.) What was the main thing that helped you decide to take part?
13.) Did you have any reservations or concerns about taking part in the study? If so, what were they?
14.) How would you describe the experience of consenting to the CALIBRE study?
15.) Who took consent? What is their role/profession?
16.) Do you know what random allocation is, how it is achieved, and what it means for your participation in CALIBRE?

Understandings of CALIBRE and treatment options

17.) What do you think the aims of the study are?
18.) Are you aware of what the two treatment options are aiming to achieve?
19.) Were you aware of VBL/Carvedilol as a possible treatment option before being introduced to the CALIBRE study?
20.) Do you feel you have an understanding of what each treatment option involves? If so, could you briefly explain this from your perspective?
21.) Did you have a preference for what treatment you are assigned to? If so, what were you reasons for preferring that treatment?
22.) a) [If randomised to preferred option] would you have considered withdrawing from the trial if you were not assigned to your preferred treatment option? Why? – Probe around the specifics of the relevant treatment
b) [If randomised to less desired preference] what made you want to continue in the trial after being randomised to your less preferred treatment option? –Probe around the specifics of relevant treatment
23.) a) How do you feel about taking carvedilol daily during the study? –Probe around side effect concerns, adherence, any support needed, and anticipated benefits, do these mitigate concerns? b) How do you feel about VBL surgery? – Probe around concerns about the surgery itself, repeated nature, recovery, risk factors and potential benefits, do these anticipated benefits mitigate concerns?
24.) Are you aware of other treatments for your condition? If so what are they?

25.) What might encourage patients to participate? – Probe around information re evidence base for treatments, improving care for those in the future, taking control over condition, clinician enthusiasm, research team accessibility/meeting, being informed of results
26.) What might put patients off taking part in the study? – Probe around Wellbeing/ill health, sharing personal information, stigma around condition, lack of clinician enthusiasm for study
27.) Is there anything we could do differently to encourage patients to take part in the study?

28.) Do you know what the follow up process for your arm of the study involves? – Probe around 4 week clinician follow up, interview between 6-12 month, CFR completion etc…

29.) What do you think is an appropriate way of measuring or understanding how the treatment has impacted upon your health?
30.) What measures of health impact are important to you personally? – Probe around lifestyle, symptoms, wellbeing and quality of life
31.) What are you expecting from involvement in the CALIBRE study?

32.) That's all the questions I had for you. Before I turn the recorder off, is there anything I haven't covered that you would like to discuss?

Supplemental A

Closing comments

Everything discussed today is completely confidential and will be untraceable to you. Having said that is there anything we discussed that you would like to withdraw? Was there anything you would like to seek clarification on? What is the best way to contact you going forward? Thank you for your time today. I will be in contact to arrange a follow up interview in the near future.

Interview Schedule- CALIBRE Phase 2, 6 month patient follow up.

Introduction, opening remarks, and consent

5.) Introduce self and tell participant you will begin recording
6.) Achieve informed consent verbally, take participant through information sheet and consent form, ensuring participant is aware of the broad aims of the study, and their rights as research participants. Fill in consent form on their behalf. Keep a copy on file and post a copy to participant.
7.) Explain the value of the voice and experience of the participant to the aims of the study.
8.) Ensure the participant is comfortable to begin the interview and give them the opportunity to ask any questions before beginning.
9.) Briefly orientate participant to topics you would like to discuss – catch up about their health, experience in the trial

Patient health

33.) Are you still on the same treatment for your varices as when you started in CALIBRE and we last spoke 6 months ago?
34.) If no, what other treatment have you received for your varices?
35.) If crossed over within trial: What were the reasons for being transferred to the other treatment?
36.) How have you been feeling over the past 6 months?
37.) Have you been experiencing any negative symptoms as a result of the treatment you have been receiving on CALIBRE? - Probe around any Adverse events (Gastro upset, Dysphagia, hypotension requiring treatment, Dysphagia)
38.) If so, did these symptoms/events get treated and/or reported back to the trial team at the hospital?
39.) Have any of these symptoms initially arose post check-up?
40.) How frequent are you experiencing these symptoms (if described)
41.) What impact are these symptoms having on your wellbeing/QOL?
42.) Are there any other changes to your circumstances or health that are relevant to the study?

Trial process experience

1.) How have you been feeling about your ongoing involvement in the trial?
2.) What have you been asked to do as a participant on the trial and what has your experience of this been? – Probe around endoscopy, banding, tolerance, recovery, impact on lifestyle, adverse event review, check-up, 5-D questionnaire (VBL). Probe around dose, tolerance, adherence, adverse event review, follow up, 5-D questionnaire, impact on lifestyle (CALIBRE). – Note, do not ask about probes if already picked up

Supplemental A

3.) Have you ever considered withdrawing from the trial? If so, what were the reasons for this? Why did you decide to continue your participation?
4.) Do you feel the 4 week check-up was useful? Probe around timing, convenience, interest.
5.) Are there any further assessments you feel we should be doing?
6.) Has the trial matched, fell below, or exceeded your initial expectations thus far? What factors have led you to come to this conclusion?
7.) How do you feel about the treatment you have been allocated to now you have experienced of it? – Probe around satisfaction
8.) Would you have preferred to have been randomised to the alternative treatment pathway?
9.) Given your experience what do you think the facilitators and barriers to patient participation in CALIBRE are?
10.) Is there anything we can do to improve the trial experience for patients?

Closing comments

Everything discussed today is completely confidential and will be untraceable to you. Was there anything you would like to seek clarification on? What is the best way to contact you going forward? Thank you for your time today.

<u>Interview Schedule- Patient Decliners</u>

Introduction and opening remarks

10.) Introduce self and tell participant you will begin recording
11.) Achieve informed consent verbally, take participant through information sheet and consent form, ensuring participant is aware of the broad aims of the study, and their rights as research participants. Fill in consent form on their behalf (if telephone interview). Keep a copy on file and post a copy to participant.
12.) Explain the value of the voice and experience of the participant to the aims of the study.
13.) Ensure the participant is comfortable to begin the interview and give them the opportunity to ask any questions before beginning.

Background of participant, context

11.) May I first ask how old you are?
12.) Can you tell me a bit about your personal background? - Probe around where from originally and react to what information participant divulges.
13.) Are you currently working?
14.) Who are the important people in your life? - Probe around family, friends if required.
15.) When did you first start to notice there may be issues with your health?
16.) What were your initial symptoms? How did they develop?
17.) Did these symptoms impact upon your daily life? If so at what point and how? – Probe around social life, relationships, working life, activities of daily living
18.) At what point did you decide to seek medical help? – Probe around time period since initial symptoms, time relevant to present day and possible catalyst/motivation for seeking help.
19.) What helped you make the decision to seek medical help?
20.) At what point were you formally diagnosed with LC?
21.)

Supplemental A

Recruitment, participation and trial process

43.) Who introduced you to the study, or how did you find out about it?
44.) How do you understand what the study involves?
45.) Were you supplied with 2 information sheets for the study?
46.) At what point did you read the PIS(s)?
47.) What did you think about the PIS(s) – Probe around understanding, accessibility, size and language used
48.) Are there any ways you feel we could improve the PIS(s)?
49.) Do you feel you were presented with enough information, to understand the study and what it, involves?
50.) Do you feel you had enough time to fully understand what the study involves before declining to participate?
51.) Was it a difficult decision to decline the study? – Probe around length of time taken to decline to participate and influence of close friends/family in decision making process
52.) What was the main thing that helped you decide to decline?
53.) How were your reservations or concerns about taking part in the study?
54.) Do you know what random allocation is, how it is achieved, and what it would have meant for your participation in CALIBRE?

Understandings of CALIBRE and treatment options

55.) Are you aware of what the two treatment options are aiming to achieve?
56.) Were you aware of VBL/Carvedilol as a possible treatment option before being introduced to the CALIBRE study?
57.) Do you feel you have an understanding of what each treatment option involves? If so, could you briefly explain this from your perspective?
58.) Did you have a preference for a treatment? If so, what are your reasons for preferring that treatment?

59.) What might have encouraged you to consent and participate in CALIBRE? – Probe around information re evidence base for treatments, improving care for those in the future, taking control over condition, clinician enthusiasm, research team accessibility/meeting, being informed of results
60.) Are there any other factors we have not already discussed that might put patients off taking part in the study? – Probe around Wellbeing/ill health, sharing personal information, stigma around condition, lack of clinician enthusiasm for study
61.) Is there anything we could do differently to encourage you to take part in the study?

62.) That's all the questions I had for you. Before I turn the recorder off, is there anything I haven't covered that you would like to discuss?

Closing comments

Everything discussed today is completely confidential and will be untraceable to you. Having said that is there anything we discussed that you would like to withdraw? Was there

Supplemental A

anything you would like to seek clarification on? What is the best way to contact you going forward? Thank you for your time today.
